# Older patients referred for geriatric consultation in the emergency department: characteristics and healthcare utilization

**DOI:** 10.1186/s12877-023-04321-2

**Published:** 2023-10-10

**Authors:** Mariangela Gagliano, Christophe J. Bula, Laurence Seematter-Bagnoud, Carole Michalski-Monnerat, Sylvain Nguyen, Pierre-Nicolas Carron, Cédric Mabire

**Affiliations:** 1 Service of Geriatric Medicine and Geriatric Rehabilitation, Lausanne University Hospital and University of Lausanne, Chemin de Mont Paisible 16, Lausanne, CH-1011 Switzerland; 2 Department of Geriatrics, Rehabilitation and Palliative Care, Neuchâtel Hospital Network, Rue du Chasseral 20, La Chaux-de-Fonds, CH-2300 Switzerland; 3 Center for Primary Care and Public Health (Unisanté), University of Lausanne, Route de la Corniche 10, Lausanne, CH-1010 Switzerland; 4 Department of Internal Medicine, Neuchâtel Hospital Network, Rue de la Maladière 45, Neuchâtel, CH-2000 Switzerland; 5 Institute of Higher Education and Research in Healthcare-IUFRS, Lausanne University Hospital and University of Lausanne, Route de la Corniche 10, Lausanne, CH-1010 Switzerland; 6 Emergency Department, Lausanne University Hospital and University of Lausanne, Rue du Bugnon 46, Lausanne, CH-1011 Switzerland

**Keywords:** Geriatric emergency medicine, Geriatric consultation, hospital admission, Older patients, Comprehensive geriatric assessment, Functional status, 30-day readmission

## Abstract

**Background:**

Comprehensive geriatric assessment (CGA) is difficult to perform in the emergency department (ED) environment and performance of screening tools in identifying vulnerable older ED patients who are best candidates for a geriatric consultation remain questionable.

**Aim:**

To determine the characteristics of older patients referred for a geriatric consultation by ED staff and to investigate these patients’ subsequent healthcare utilization.

**Methods:**

Secondary analysis of data previously collected for a prospective observational study of patients aged 75 + years visiting the ED of an academic hospital in Switzerland over four months (Michalski-Monnerat et al., J Am Geriatr Soc 68(12):2914–20, 2020). Socio-demographic, health, functional (basic activities of daily living; BADL), cognitive, and affective status data were collected at admission by a research nurse using a standardized brief geriatric assessment. Information on geriatric consultations, hospitalization, discharge destination, and 30-day readmission were retrieved from hospital database. Bivariable and multivariable analyses were performed using this data set collected previously.

**Results:**

Thirty-two (15.8%) of the 202 enrolled patients were referred for a geriatric consultation. Compared to the others, they were older (84.9 ± 5.4 vs 82.9 ± 5.4 years, *p* = .03), more impaired in BADL (4.8 ± 1.6 vs 5.5 ± 1.0, *p* = .01), with more comorbid conditions (5.3 ± 1.5 vs 4.5 ± 1.9, *p* = .03), more frequently admitted after a fall (43.7% vs 19.4%, *p* = .01), and hospitalized over the previous 6-month period (53.1% vs 30.6%, *p* = .02). Multivariable analyses that adjusted for variables significantly associated with outcomes in bivariable analysis found that being admitted after a fall (AdjOR 4.0, 95%CI 1.7–9.4, *p* < .01) and previously hospitalized (AdjOR 2.7, 95% CI 1.2–6.2, *p* = .02) remained associated with increased odds of consultation, whereas the inverse association with BADL performance remained (AdjOR 0.7, 95%CI 0.5–0.9, *p* = .01).

Patients referred for geriatric consultation had higher odds of hospitalization (84.4% vs 49.4%; AdjOR 5.9, 95%CI 2.1–16.8, *p* < .01), but similar odds of home discharge when admitted, and of 30-day readmission.

**Conclusion:**

About one in six older ED patients were referred for a geriatric consultation who appeared to be those most vulnerable, as suggested by their increased hospitalization rate. Alternative strategies are needed to enhance access to geriatric consultation in the ED.

**Supplementary Information:**

The online version contains supplementary material available at 10.1186/s12877-023-04321-2.

## Introduction

The proportion of older patients visiting the Emergency Department (ED) is increasing in western countries [1–6] and, among older patients, those aged 85 years and over have twice the rate of ED visits than their younger counterpart [5, 7]. Indeed, these older patients often suffer from multiple diseases, from functional and cognitive impairments, entangled with psychosocial issues that all concur to increase their risk of ED use. Due to their vulnerability, these older patients are also exposed to a greater risk of adverse events once hospitalized [8–10].

ED international guidelines increasingly recommend adapting ED care delivery process to older patients [11–15]. In particular, the identification of vulnerable older patients at risk for adverse events when hospitalized is now strongly recommended to improve their subsequent management and orientation in the healthcare maze [16, 17]. Comprehensive geriatric assessment (CGA) has been identified as a potentially useful approach to this aim as it was shown effective to improve functional trajectories and the likelihood of remaining at home up to 12 months after discharge from dedicated geriatric wards [9];[18, 19]. Indeed, several studies that specifically examined CGA-based geriatric consultations performed by teams working within EDs reported promising results in detecting vulnerability and preventing admission [20–22]. However, evidence from these studies was less clear about their effect on length of stay or 30-day readmission [20–22]. Additional uncertainties about this approach result from difficulties in performing CGA in the busy ED environment, as well as questionable performance of screening tools in identifying vulnerable older ED patients who are best candidates for a geriatric consultation [23–26]. Indeed, to our knowledge, studies that investigated patients’ characteristics associated with referral for a geriatric consultation in the ED are scarce [27]. Uncertainty remains regarding how best to identify older patients at increased risk of complex health care trajectories, including 30-day hospital readmission [23, 28–31], even though recent studies highlight the importance of functional, cognitive, as well as social status as major determinants of older ED patients’ risk to be admitted to the hospital [32].

A two-step approach was implemented at the ED of Lausanne University Hospital. A list of “red flags”, inspired by the “Geriatric 5Ms” (Supplementary Table 1) [15, 33] was initially implemented when the geriatric team started consulting in the ED. This list was proposed to assist ED staff in identifying older vulnerable adults who might then benefit from a CGA performed by the geriatric consultation team. This list was primarily used to raise ED staff awareness to the need of screening of geriatric vulnerabilities in ED according to international recommendations rather than for pre-triage purpose. The ED staff is however free to refer any older patient for a geriatric consultation according to his own assessment.

The main objective of this study was to determine the characteristics of patients referred by the ED staff for a geriatric consultation. Specifically, we wanted to explore whether these characteristics would be congruent with the red flags currently used in our hospital setting.

A secondary objective was to investigate whether ED patients referred for a geriatric consultation differed from other older ED patients in subsequent healthcare utilization. Specifically, we hypothesized that patients with a geriatric consultation in the ED would have a) lower rates of hospital admission after the ED visit; b) higher rates of home discharge when admitted; c) lower rates of readmission 30 days after the initial ED visit [20].

## Methods

This study is a secondary analysis of data previously collected in a prospective cohort study that investigated the predictive performance of the interRAI Emergency Department Screener (EDS) [23].

### Population

Eligible patients were those aged 75 years or older who presented over a 4-month period (from October 1, 2018 to January 31, 2019) to the ED of an academic hospital in Switzerland during weekly daytime. Patients with life-threatening conditions according to the Swiss Triage Scale (i.e., score = 1) [34] and those unable to communicate in French or to sign an explicit consent were excluded. A total of *N* = 202 participants who completed the brief geriatric assessment were included in the original study. This sample size was calculated for the original study, based on an estimated 60% hospital admission rate and targeting a sensitivity of 90% and an accuracy of 5% as proposed in the Standards for Reporting Diagnostic Accuracy Studies (STARD) checklist [23].

### Measurements

A dedicated research nurse collected data on socio-demographic (including age, sex, living situation, presence of a caregiver) and performed a standardized brief geriatric assessment [35] using validated instruments to screen selected dimensions including: polypharmacy (taking five or more drugs per day), gait impairment (self-reported fall in the previous 2-month period, use of a walking aid), risk of malnutrition (weight loss ≥ 5% in the last 3 months), functional (BADL[36], IADL [37], cognitive (MiniCog, [38], CAM [39]), and affective (miniGDS [40]) status at the time of patients’ ED visit Information on health conditions and hospitalization in the previous 6-month period were retrieved from patients’ electronic health records (EHR) and the hospital administrative database, respectively.

### Outcome measures

The occurrence of a geriatric consultation in the ED was determined from a systematic review of the hospital EHR of all included patients. Data on patients’ healthcare utilization after the index ED visit were retrieved from the hospital administrative database. Hospital admission after the index ED visit and 30-day readmission (ED or hospital) were determined for the entire sample. Discharge destination was determined in those admitted to the hospital after their index ED visit.

### Statistical analysis

Usual statistics (proportion, means, median) were used to describe the population. To compare characteristics of patients with and without a geriatric consultation in the ED, parametric (Student’s t, Chi2) and non-parametric (Kruskal–Wallis, Fisher exact) tests were used, depending on data distribution. A multivariable logistic regression analysis was performed to predict a geriatric consultation (adjusted OR; AdjOR). Candidate variables included in the multivariable model were those significantly associated with a geriatric consultation in bivariable analysis.

Bivariable analysis and multivariable logistic regression analyses were also performed to determine the association between the occurrence of a geriatric consultation and each specific secondary outcome (hospital admission; discharge destination; 30-day readmission). Covariates included in each model were also determined from results of bivariate analyses. All analyses were performed with STATA (Stata Statistical Software: Release 17. College Station, TX: StataCorporation. 2021).

## Results

### Characteristics of patient’s referred for a geriatric consultation in the ED

Overall, 32 (15.8%) of the 202 patients included in the study were referred for geriatric consultation by the ED staff. Compared to those without consultation (Table 1), these patients were older (84.9 ± 5.4 vs 82.9 ± 5.4 years, *p* = 0.03), more impaired in BADL (4.8 ± 1.6 vs 5.5 ± 1.0, *p* = 0.01), more frequently taking five or more medications (84.4% vs 61.2%, *p* = 0.01), and had more comorbid conditions (5.3 ± 1.5 vs 4.5 ± 1.9, *p* = 0.03). In addition, they were also more frequently admitted after a fall (43.7% vs 19.4%, *p* = 0.01), and hospitalized in the previous 6-month period (53.1% vs 30.6%, *p* = 0.02).

**Table 1:** Characteristics of the study population and their comparisons in patients with and without a geriatric consultation in the Emergency Department (ED)

Characteristics	Total	Benefited from a geriatric consultation?		*P* value^*^
	(*N* = 202)	**Yes** (*N* = 32)	**No** (*N* = 170)	
**Age (mean, SD)**	83.2 (5.4)	84.9 (5.4)	82.9 (5.4)	.03
**Gender (male)**	87 (43.1%)	10 (31.2%)	77 (45.3%)	.17
**Living alone**	88 (43.6%)	18 (56.2%)	70 (41.2%)	.12
**Caregiver present in ED**	47 (23.3%)	7 (21.9%)	40 (23.5%)	1.00
**Admitted for a fall**	47 (23.3%)	14 (43.7%)	33 (19.4%)	.01
**Hospitalized in the last 6-month**	69 (34.2%)	17 (53.1%)	52 (30.6%)	.02
**Delirium**^**a**^	7 (3.5%)	2 (6.2%)	5 (2.9%)	.31
**Cognitively impaired**^**b**^	98 (49.0%)	16 (51.6%)	82 (48.5%)	.84
**Depressive symptoms**^**c**^	77 (38.1%)	15 (40.9%)	62 (36.5%)	.32
**Denutrition**	93 (46.0%)	16 (50.0%)	77 (82.8%)	.79
**Polymedication > 5**	131 (64.8%)	27 (84.4%)	104 (61.2%)	.01
**Comorbidity (mean, SD)**	4.7 (1.8)	5.3 (1.5)	4.5 (1.9)	.03
**Number of treatment (mean, SD)**	6.8 (0.3)	7.9 (0.7)	6.6 (0.3)	.11
**Basic ADLs score**^**d**^** (mean, SD)**	5.4 (1.2)	4.8 (1.6)	5.5 (1.0)	.01

*Abbreviations ADL* Activities of daily living, *SD* Standard deviation, *ED* Emergency department

^a^According to the Confusion Assessment Measure (CAM) [39]

^b^According to Minicog [38]

^c^According to 4-item Geriatric Depression Scale (GDS) [40]

^d^From Katz’s basic activities in daily living (ADLs) [36]; scores range from zero to six, with higher scores indicating higher independence

^*^P-value from Student’s t-test, Chi squared test, or Fisher exact test, depending on the type of variables and its distribution

**Fig. 1:**
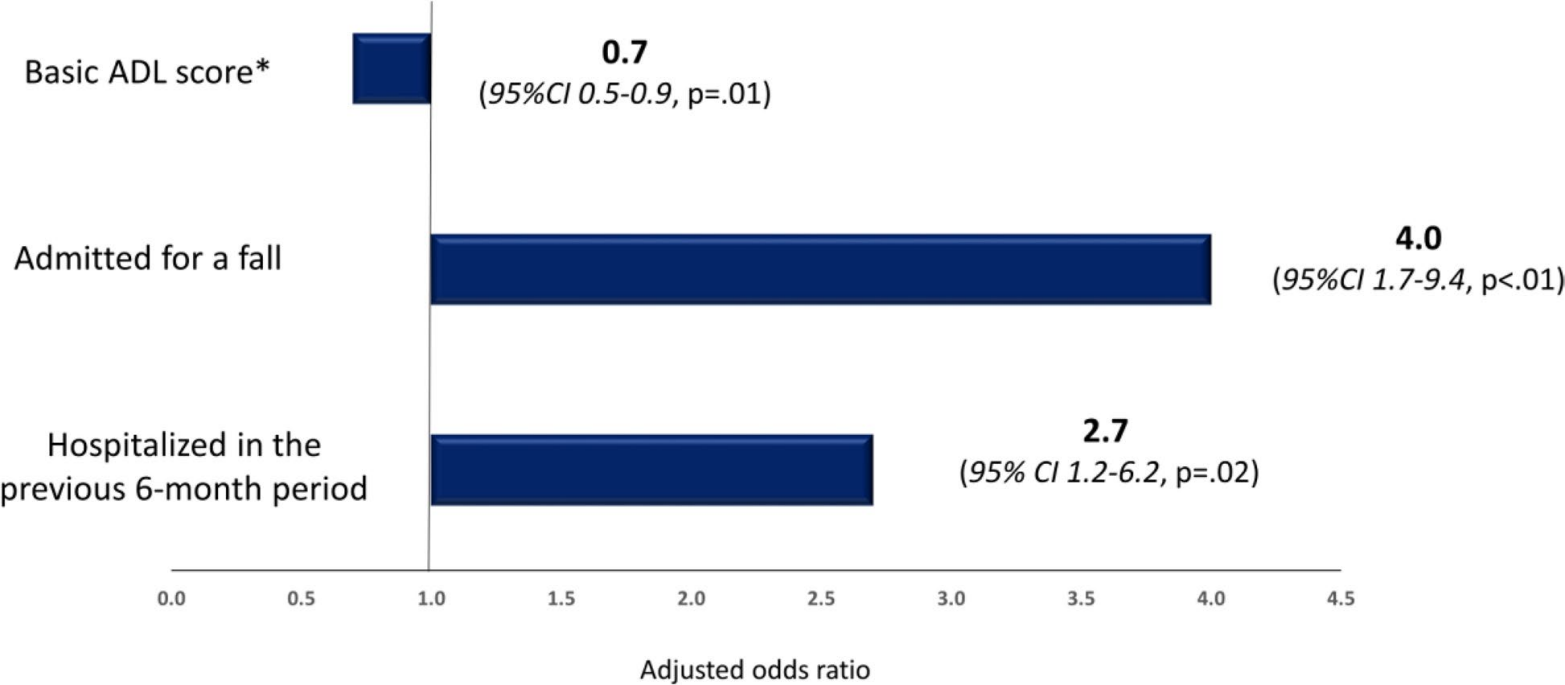
Results of multivariable analysis of patients’ characteristics associated with the occurrence of a geriatric consultation in the Emergency Department (Adjusted odds ratio from multivariable logistic regression). * Basic ADL score: score at Katz’s basic activities of daily living scale; range from 0 to 6 with higher score indicating better performance

In multivariable analysis (Fig. 1), being admitted after a fall (AdjOR 4.0, 95% CI 1.7–9.4, *p* < 0.01) and being hospitalized in the previous 6-month period (AdjOR 2.7, 95% CI 1.2–6.2, *p* = 0.02) remained associated with higher odds to be referred for geriatric consultation in the ED. In contrast, an inverse association remained with BADL performance (AdjOR 0.7, 95%CI 0.5–0.9, *p* = 0.01). All three characteristics are included in the list of “red flags” currently used in our hospital setting (Supplementary Table 1).

#### Healthcare utilization

**Fig. 2:**
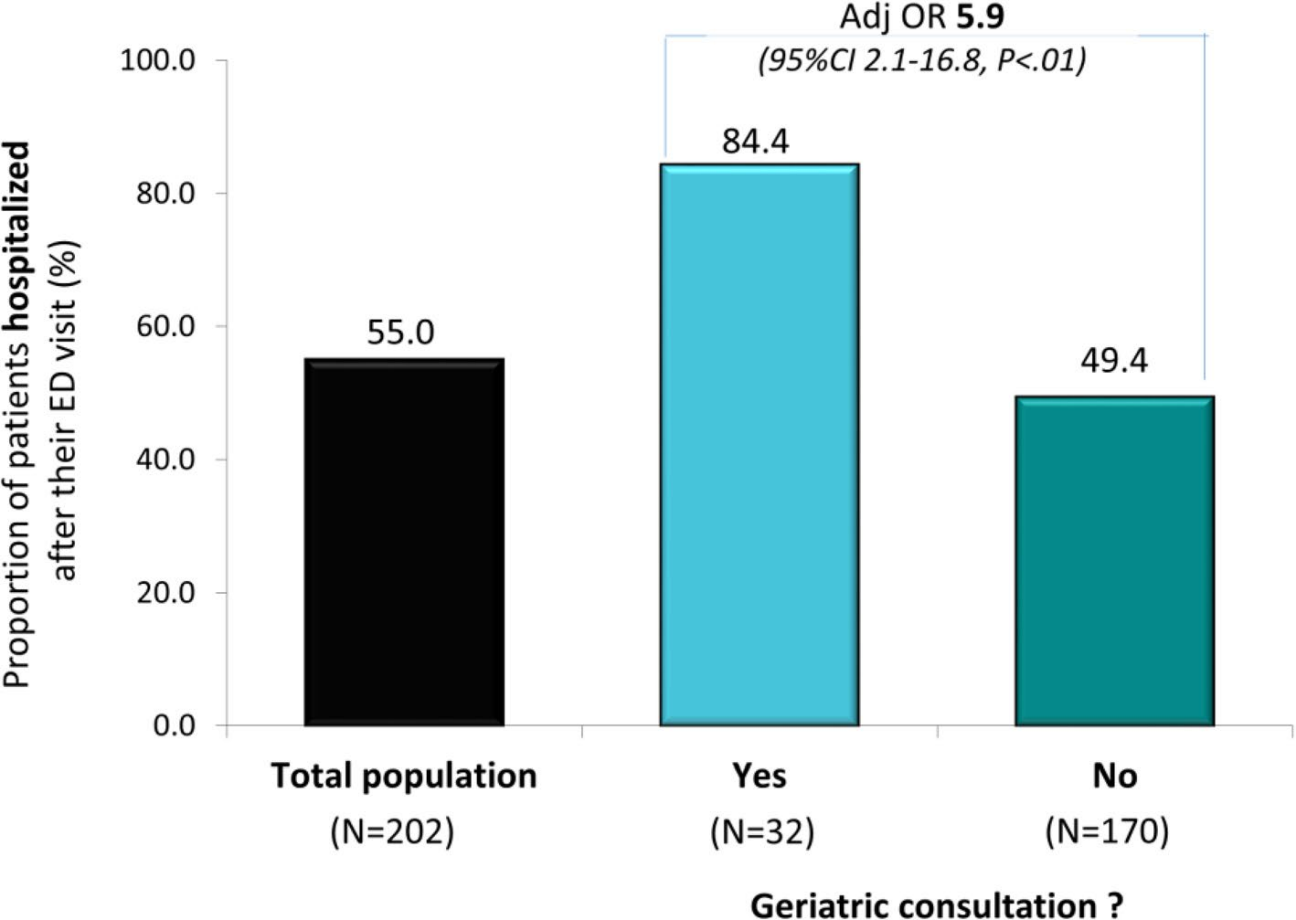
Proportion of patients hospitalized after their ED visit in the total population, and in those with and without a geriatric consultation (AdjOR: adjusted Odds Ratio; 95% CI: 95% confidence intervals) adjusted for falls admitting diagnosis, performance in basic ADLs, and hospital admission in the previous 6-month period

Overall, 111 (55.0%) of the 202 included patients were admitted to the hospital after their index ED visit (Supplementary Table 2). All seven patients with delirium were admitted. The only other characteristic that differed between patients admitted or not was the occurrence of a geriatric consultation that occurred in 24.3% of hospitalized patients vs only 5.5% (*p* = 0.01) of those not admitted. Indeed, 84.4% of patients with a geriatric consultation were hospitalized as compared to 49.4% of those without a consultation (OR 5.5, 95%CI 2.0–15.0, *p* < 0.01). This association remained in multivariable analysis (AdjOR 5.9, 95%CI 2.1–16.8, *p* < 0.01) that adjusted for functional status, a fall admitting diagnosis, and previous hospital admission (Fig. 2).

**Fig. 3:**
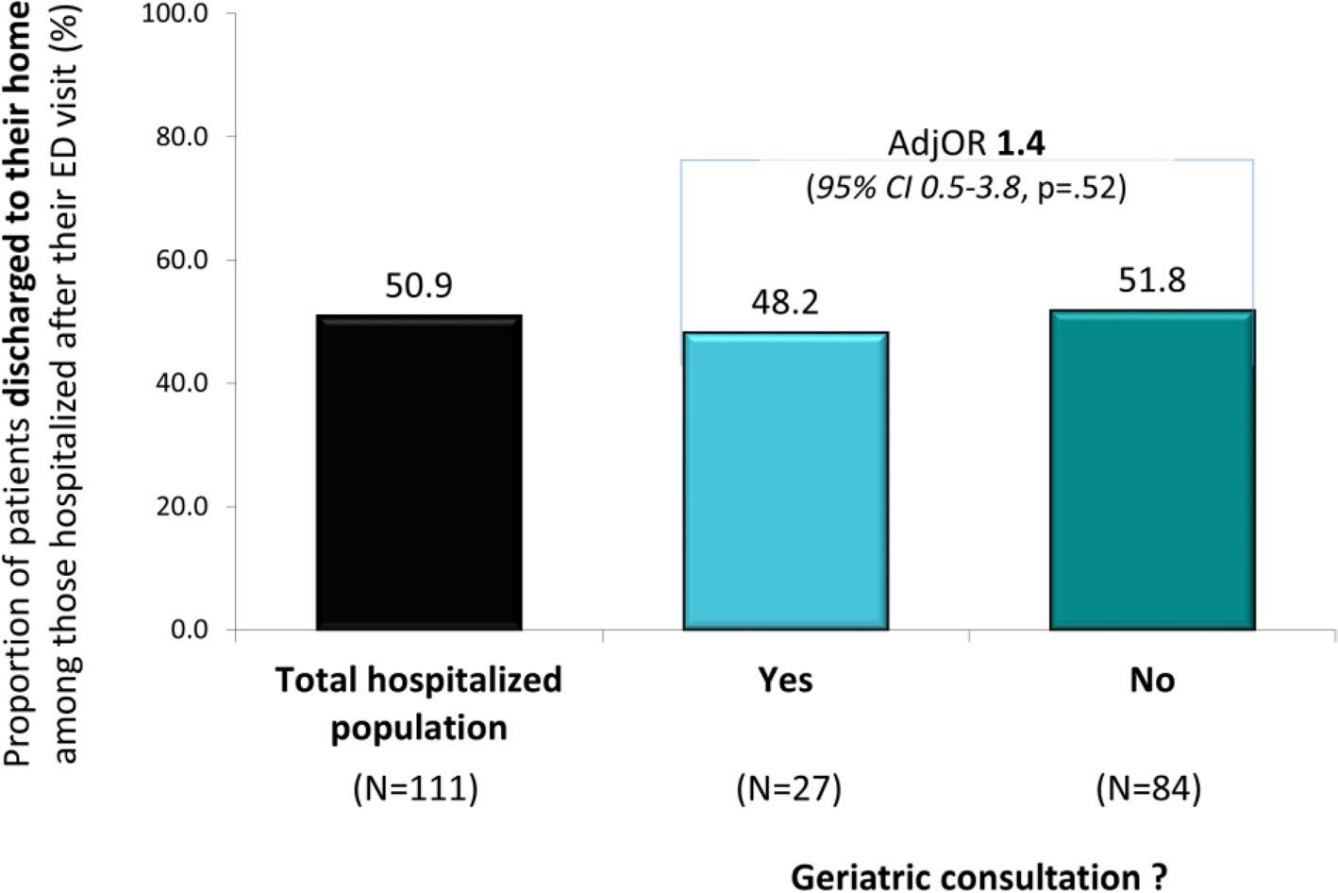
Proportion of patients discharged to their home after their hospitalization in the total population, and in those with and without a geriatric consultation (AdjOR: adjusted Odds Ratio; 95% CI: 95% confidence intervals) adjusted for age, living alone, presence of delirium, and cognitive status)

Among patients admitted (*N* = 111), about half (50.9%) were discharged directly to their home (Fig. 3). This proportion did not differ among patients with and without a geriatric consultation (48.2% vs 51.8%; AdjOR 1.4, 95%CI 0.5–3.8, *p* = 0.52).

**Fig. 4:**
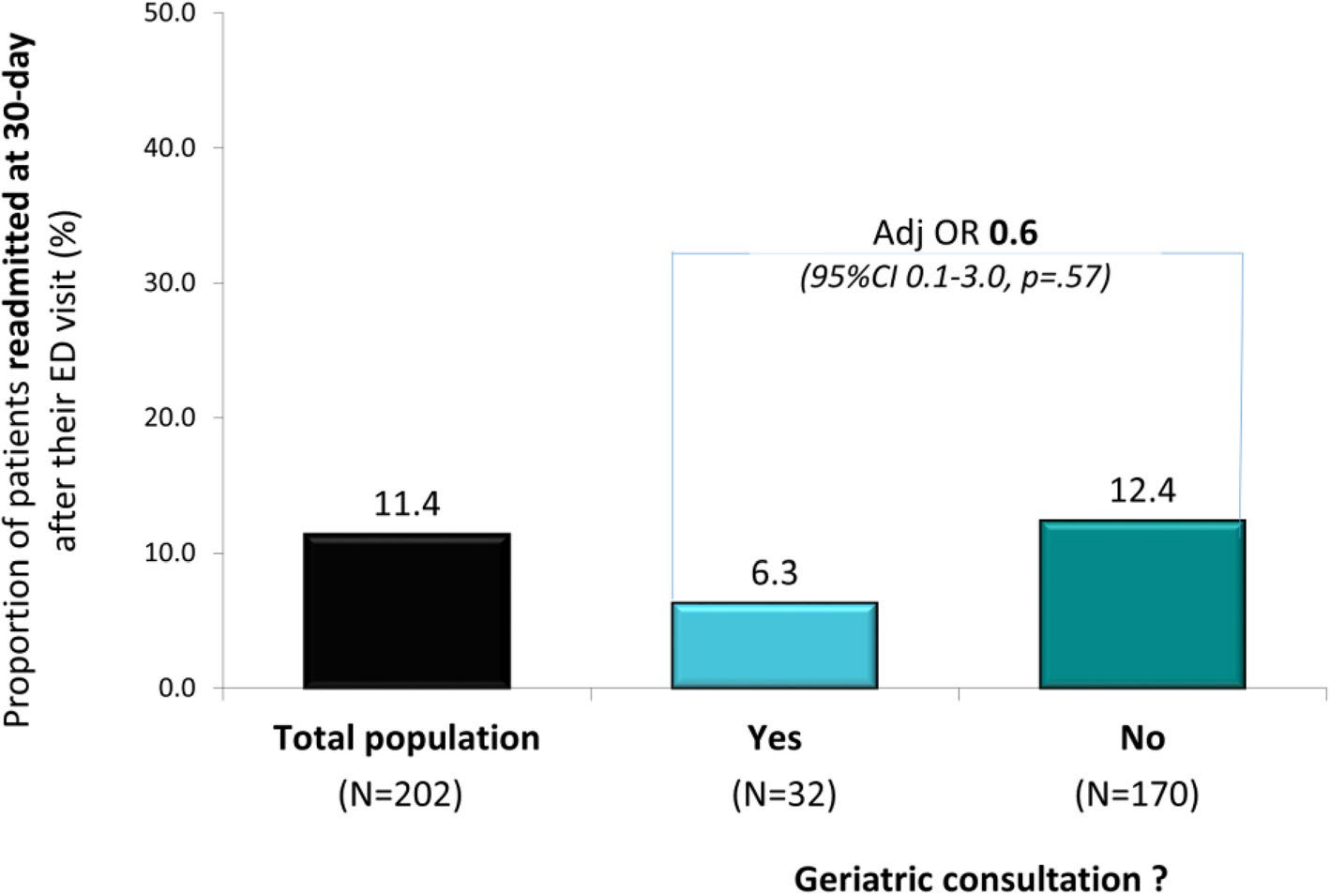
Proportion of patients readmitted at 30-day after their initial ED visit in the total population, and in those with and without a geriatric consultation (Adj OR: Adjusted Odds Ratio; 95% CI: 95% confidence intervals); adjusted for performance in basic ADLs

Finally, only 23 (11.4%) patients were readmitted within 30 days (Fig. 4). Those who were referred for geriatric consultation were twice less frequently readmitted at 30-day follow-up, but this difference did not reach statistical significance (6.3% vs 12.4%, AdjOR 0.6, 95%CI 0.1–3.0, *p* = 0.57).

## Discussion

This study shows that, in this convenience sample, only about one in six older patients visiting the ED were referred for a geriatric consultation. This sobering finding is somewhat tempered by results showing that patient’s characteristics associated with the occurrence of these consultations closely concur with the “red flags” characteristics as defined to target them. These contrasted results are important from several perspectives. First, they suggest that access of older ED patients to geriatric competencies and expertise still remains limited, even in an ED environment with a genuine interest in improving care for this population. These findings could also be interpreted as an illustration of the current shortage in ED geriatric resources. Overall, these results encourage to consider alternative strategies to enhance access to geriatric expertise in the ED. Unfortunately, screening instruments have shown less than satisfactory performance in this setting, as recently reported [23, 24]. Finally, these results extends previous observations in showing that patient’s characteristics identified in the current study are a mix of factors related to functional, mobility, and previous health problems, thus landing further support for the use of CGA-based approach to address these patients’ needs.

The observation that patients referred for geriatric consultation were more frequently admitted to the hospital is in contrast with results of previous interventional studies and differs from our initial hypothesis [20]. Several explanations could be proposed that are not mutually exclusive. First, it is possible that admissions were increased because geriatricians identified conditions that might not have been diagnosed in the absence of a geriatric consultation. Alternatively, older patients referred in priority by the ED staff might be those selected because they present red flags criteria, are most vulnerable and already at high risk to be admitted. Noteworthy, these patients had more frequently been hospitalized in the previous 6 months. Another hypothesis could be the lack of existing alternatives to acute hospitalization for older patients in the healthcare environment, (e.g., absence of a hospital-at-home program) or the shortage of short-term beds in nursing homes. [41] Finally, a less likely explanation could be that results from the geriatric consultation raised unnecessary worries in the medical team, resulting in inappropriate admissions. This would need to be further investigated.

Our initial hypothesis that older ED patients referred for a geriatric consultation would be less likely to be readmitted at 30-day was not confirmed, even though the proportion of patients readmitted was twice lower among these patients than in those without geriatric consultation. Unfortunately, the small number of readmissions limit the study’s statistical power and precludes any firm conclusion. Larger studies will be needed to further investigate this important outcome.

The main limitation of this study is its relatively small sample size that resulted in a too low statistical power for some secondary outcomes (i.e., 30-day readmission). The convenience sampling with exclusion of unstable patients, those unable to sign the informed consent, and those who did not complete the brief geriatric assessment in the design of the previous study, [23] all limit the generalizability of this study’s findings. In particular, this likely explains the limited proportion of patients with delirium observed in this study (3.5%), a prevalence much lower than the 8% to 17% usually observed in other ED studies [42, 43]. Finally, the study was performed in a single center, within a specific ED setting and healthcare environment, and generalization of findings should be cautious.

## Conclusion

About one in six older patients visiting the ED were referred for a geriatric consultation. Referred patients were more vulnerable, frequently hospitalized within the previous 6-month period, admitted after a fall, and had lower performance in basic ADL, all characteristics fully consistent with the “red flags” characteristics as defined to target them. These results could certainly be useful in developing further studies about triage of ED vulnerable patients. In addition, these observations further support a CGA-based approach to better address these patients’ needs. Patients referred for a geriatric consultation were more frequently hospitalized after their ED visit, strongly suggesting a selection bias. Future studies should further investigate whether this observation results from a possible less frequent use of red flags by the ED staff in patients rapidly discharge to their home and/or a more frequent use in patients felt to be likely candidate to hospital admission. Although subsequent healthcare utilization did not differ in patients with and without geriatric consultations, the non-significant difference in 30-day readmission rates across the two groups deserves to be further investigated in larger sample.

### Supplementary Information

**Additional file 1: Supplementary Table 1. **“Red flags” used at Emergency Department (ED) to identify older patients who are candidates for a geriatric consultation. **Supplementary Table 2. **Comparisons of characteristics of patients hospitalized or not after their Emergency Department (ED) visit.

## Data Availability

Data are not publicly available but could be requested from the corresponding author.
