# Supplementary material for: Older patients referred for geriatric consultation in the emergency department: characteristics and healthcare utilization
Source: BMC Geriatr. 2023 Oct 10;23:642. doi: 10.1186/s12877-023-04321-2 (PMC10565963; doi:10.1186/s12877-023-04321-2)
Supplement: Supplementary file 1 — Additional file 1: Supplementary Table 1. “Red flags” used at Emergency Department (ED) to identify older patients who are candidates for a geriatric consultation. Supplementary Table 2. Comparisons of characteristics of patients hospitalized or not after their Emergency Department (ED) visit. [file 12877_2023_4321_MOESM1_ESM.pdf]

Supplementary Table 1. “Red flags” used at Emergency Department (ED) to identify older patients who are candidates for a geriatric consultation.

Aged ≥ 85 years

Aged ≥ 65 years with at least one of the following:

- Delirium at the time of ED admission
- Dementia diagnosis / Cognitive decline / Cognitive impairment
- Functional limitations: dependency in Basic Activities of Daily Living (BADL)
- Mobility impairment: admitted for a fall ; gait and/or balance impairment; history of a fall in the previous 12-month period
- Hospitalization in the previous 3-month period
- Social isolation: living alone; difficulties in home care; caregiver burnout

Supplementary Table 2. Comparisons of characteristics of patients hospitalized or not after their Emergency Department (ED) visit.

Characteristics	Total	Hospitalized after the ED visit?		*P* value*
	(n=202)	Yes (n=111)	No (n=91)	
**Geriatric consultation (n, %)**	32 (15.8%)	27 (24.3%)	5 (5.5%)	.01
**Age (mean, SD)**	83.2 (5.4)	83.6 (5.3)	82.6 (5.6)	.20
**Gender (male) (n, %)**	87 (43.1%)	50 (40.5%)	37 (46.6%)	.53
**Living alone (n, %)**	88 (43.6%)	46 (41.4%)	42 (46.2%)	.50
**Caregiver present in ED† (n, %)**	47 (23.3%)	26 (23.4%)	21 (23.1%)	.95
**Admitted for a fall (n, %)**	47 (23.3%)	25 (22.5%)	22 (24.2%)	.78
**Hospitalized in the last 6-month (n, %)**	69 (34.2%)	41 (36.9%)	28 (30.8%)	.36
**Delirium †**	7 (3.5%)	7 (6.3%)	0 (0.0%)	.02
**Cognitively impaired ‡**	98 (49.0%)	54 (49.5%)	44 (48.4%)	.87
**Depressive symptoms §**	77 (38.1%)	45 (40.6%)	32 (35.2%)	.43
**Basic ADL score ‖ (mean, SD)**	5.4 (1.2)	5.3 (1.3)	5.5 (0.9)	.23

* From Student's t-test, Chi squared test, or Fisher exact test, depending on the type of variables and its distribution

† According to the Confusion Assessment Measure (CAM) [[36]]

‡According to Minicog [[31]]

§ According to 4-item Geriatric Depression Scale (GDS) [[32]]

‖ From Katz's basic activities in daily living (ADLs)[[30]]; scores range from zero to six, with higher scores indicating higher independence
